# A gradient-based, GPU-accelerated, high-precision contour-segmentation algorithm with application to cell membrane fluctuation spectroscopy

**DOI:** 10.1371/journal.pone.0207376

**Published:** 2018-12-06

**Authors:** Michael Mell, Francisco Monroy

**Affiliations:** 1 Mechanics of Biological Membranes and Biorheology, Dpto. Química Física I, Universidad Complutense, Madrid, Spain; 2 Translational Biophysics, Instituto de Investigacion Biomédica Hospital Doce de Octubre (i+12), Madrid, Spain; Science and Technology Facilities Council, UNITED KINGDOM

## Abstract

We present a novel intensity-gradient based algorithm specifically designed for nanometer-segmentation of cell membrane contours obtained with high-resolution optical microscopy combined with high-velocity digital imaging. The algorithm relies on the image oversampling performance and computational power of graphical processing units (GPUs). Both, synthetic and experimental data are used to quantify the sub-pixel precision of the algorithm, whose analytic performance results comparatively higher than in previous methods. Results from the synthetic data indicate that the spatial precision of the presented algorithm is only limited by the signal-to-noise ratio (SNR) of the contour image. We emphasize on the application of the new algorithm to membrane fluctuations (flickering) in eukaryotic cells, bacteria and giant vesicle models. The method shows promising applicability in several fields of cellular biology and medical imaging for nanometer-precise boundary-determination and mechanical fingerprinting of cellular membranes in optical microscopy images. Our implementation of this high-precision flicker spectroscopy contour tracking algorithm (HiPFSTA) is provided as open-source at www.github.com/michaelmell/hipfsta.

## Introduction

Quantitative imaging is progressively empowering the analytic toolbox of cellular biology with high-performance observational facilities accessing new biophysical markers resolved in space and time [1,2]. Optical microscopy imaging combined with computational technologies are opening translational promise as provide the integrative biologist with unprecedented mines of data to be exploited in high-performance analytic approaches, which are allowing to gain further insight beyond conventional imaging with immunofluorescence [3]. Fluctuation spectroscopy with optical contrast microscopes is a simple, yet powerful biophysical method to probe non-invasively mechanical properties of biological cells by imaging the shape fluctuations of the cell membrane contour [4]. When applied to living cells undergoing metabolically-driven (non-equilibrium) fluctuations, the fluctuation method has allowed for getting new insights into cell mechanics, particularly in studies with red blood cells, hereinafter RBCs [5–12]. The performance of contour-segmentation algorithms for tracking membrane fluctuations from video-microscopy images has always played a crucial role in the advance of this technique by enabling a more precise determination of the cell contour-position [13]. Accordingly, various segmentation methods have been proposed with contrast imaging [7,13–21], which were accompanied by technological advances in digital image processing and computational power. The contour-segmentation algorithm we present here harnesses the computational power of general purpose graphics processing units (GPGPU) having recently become available to significantly improve on current segmentation methods in the sub-pixel performance of nanometer resolution. The super-localization software here proposed may enable observation of novel dynamical phenomena in cell biology and may allow for significantly more precise results that could be exploited efficiently in cell phenotyping approaches useful in biomedical contexts. The new algorithm is potentially implementable to more sophisticated optical settings [1], including diffraction phase microscopy (DMP) [22] and phase-contrast modes in digital holographic microscopy (DHM) [23]. These adaptations could push quantitative imaging further into novel software developments that take advantage of the enhanced contour detection provided by our new method.

**Fig 1:**
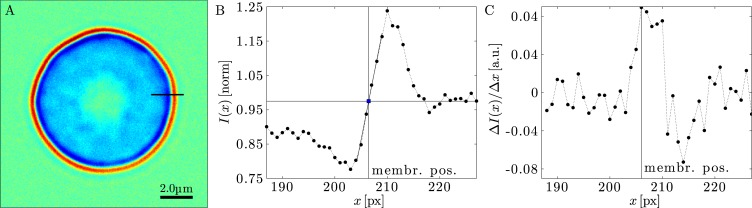
Phase contrast causes an intensity gradient at the location of the RBC membrane. (A) Bright-field image of a RBC. (B) Intensity profile along the black line in (A). The vertical line indicates the contour-position as found with the method described in [13]. The background intensity and fit are also indicated (horizontal and diagonal line). (C) Intensity-gradient of (B). The vertical line indicates the maximum. The noise in the image cause two maxima at the peak, which makes localizing the absolute peak value difficult without filtering.

Technically speaking, the new segmentation algorithm analyzes optically contrasted (either bright-field or phase-contrast) microscopy images of cells possessing a characteristic halo at the cell boundary. The so-called “halo effect” is nonlinearly tied to the coherence of the illumination and the optical contrast of the sample [24], and provides an exploitable framework for image formation in different contrast modes under varying degrees of coherence [25,26]. This contrast halo consists of an intensity minimum and maximum, which encodes the information on the membrane position as the result of a mismatch in the refractive indices of the medium inside and outside the cell (see Fig 1A and 1B). Early segmentation algorithms used the intensity maximum of the halo to determine the location of the cell-contour [14,16,19,27]. However, it was later pointed out by Döbereiner [18] and Pécréaux [13] that the location of the interface is actually placed at the maximal gradient of the halo intensity [28] and this has become the preferred method for the membrane localization [13,16–18,20,21]. Pécréaux and cols. [13] proposed the currently widespread segmentation algorithm, where the interface location is defined by the intercept of a linear fit to the intensity profile at the contour halo and the average background intensity (see Fig 1B). This linear fit is performed to the pixel-intensity values along three of the four principle directions of the pixel-grid (2x parallel, 2x diagonal) to calculate the final position from their weighted average [13]. In the Pécréaux’ algorithm segmentation is performed sequentially along the contour using a number of different conditions to determine the segmentation direction. More recently, new segmentation methods have been proposed to locate the contour positions by directly determining the maximum of the intensity gradient [20,21]. In particular, Usenik et al. [21] used interpolation to calculate the image intensity along radial directions starting from the center of the contour. The contour position is determined by the intensity gradient maximum along each individual line (see Fig 1(C)). By calculating an updated barycenter of the contour, the process is repeated for each image until the center of the contour converges to a fixed position. This allows the individual refinement of each contour-coordinate, an improvement that is not possible using previous methods. However, the direct usage of the numerical gradient in the Usenik’s method has drawbacks, since it amplifies image noise. Therefore, those authors use Gaussian image filter to reduce noise, which should be expected to reduce resolution [21].

To overcome the limitations of these two algorithms, our proposed method combines ideas from both, putting substantial emphasis on image oversampling as an original characteristic of the proposed method. In Pécréaux’ method each point in the contour is determined from the precedent one, which introduces an evident bias that makes that method especially sensible to the choice of the initial condition. To increase the robustness of our algorithm, we follow the Usenik’s method for which each contour coordinate ***P***_*i*_ is refined independently at a fixed contour angle *ϕ*_*i*_ over various iterations per image. Motivated by Pécréaux, our method determines each contour coordinate ***P***_*i*_ from the weighted sum of a large number of linear fits over a range of local angles *φ*_*j*_ centered on the contour normal ***n***_*i*_ of the intensity halo. In our proposed method, the positions for performing the linear fits become independent of the pixel-grid by interpolating pixel-intensities, which overcomes a subtle, but significant issue with Pécréaux’ method that is related with the fixed positions of the centers of the pixels. These mutual improvements have allowed us to generate a new hybrid algorithm, more robust and precise than the previous ones, which enhances the accuracy of the segmentation method down to 2nm spatial precision in determining changes in the position of the contour halo. The new algorithm has been implemented with movies of dynamic fluctuations recorded by digital video-microscopy with high-velocity cameras at short exposition times (shorter than millisecond). This allows an ultrafast readout of the contour fluctuations (up to several tents kHz) that, applied to cell membranes, allows imaging the instantaneous snapshots necessary for probing the shape fluctuations of the cell membrane. A typical imaging session left less than one-second, the time necessary to record all the data required to perform the time-average involved in a statistically relevant fluctuation spectrum and to detect the time correlations due to the intrinsic dynamics of the fluctuation modes of membrane deformation. Further mathematical treatment of these membrane fluctuations, especially by Fourier analysis, makes possible a deep understanding of membrane mechanics in terms of usual elasticity models [29]. All these improvements empower the proposed method with a significantly higher performance than previous ones, particularly in terms of enhanced spatiotemporal accuracy that allows resolving the membrane fluctuations with a higher precision not only in amplitude but also in spatial and time resolution, both in real and reciprocal domains. Such a breakthrough in segmentation performance should be crucial to approach new problems of cell mechanics where membrane fluctuations can be exploited as a relevant observable. Imaging membrane fluctuations could thus become an excellent observational method for non-invasive and non-stressing probing the mechanical phenotypic traits of cellular membranes where the intrinsic rigidity of the cytoskeleton necessarily imposes low amplitudes and short correlation times and distances of the membrane fluctuations.

## Method

### Microscopy hardware

To observe the surrounding border of a cell, or of a lipid vesicle, we take advantage of wide-field transillumination video-microscopy [30]. Transillumination techniques require two lenses, condenser and objective, both used to focus the light on each side of the sample. In an inverted bright-field (BF) microscope, the sample is transilluminated from above by focusing light (with the condenser) on the entire field of view where the object to observe is placed. The resulting BF image is formed after the light has been transmitted through the sample and collected with the objective lens from its focal plane. Then, the image is recorded on a video camera. Because of the transmittance difference between the object interior and the outer medium, an Airy pattern due to edge-diffraction is produced at the border of the object. Consequently, when the BF image is formed, the object appears surrounded by a brighter “halo”, which delimits the contour of the border [31]. BF images of our biological samples tend to have low contrast, because vesicles and most cells are not strongly light-absorbing, however additional contrast can be gained in the phase-contrast mode [30]. In a Phase Contrast (PhC) microscope, phase shifts produced in light passing through a transparent specimen with contrasted optical densities are converted to brightness changes in the image. PhC is based on refractive index differences [32], indeed optical edges between regions with different refractive indices cause light to refract in an amount (phase-shift) that depends on how much the refractive index changes. Most simply, PhC microscopes generate an image by comparing how much phase-shift is produced at each location in the sample relative to how much light is not phase-shifted. Physically, this construction occurs via interference between refracted (phase-shifted) and non-refracted light beams. The PhC microscope is fitted with a special condenser and a phase-contrast objective, which contain both phase rings to control phase contrast. PhC images may be made to appear dark against a bright background (positive contrast) or bright against a dark background (negative contrast). The borders of images are surrounded by a characteristic phase-contrast “halo”. Both, BF and PhC techniques are highly sensitive and compatible with the short exposure times involved with high frame rate recording.

The proposed contour-segmentation algorithm has been conceived to work with halo-edged contrasted microscopy images, indeed it can be interoperable with images obtainable from different transillumination modes used to generate optical contrast. Because the halo contrast is tied to the coherence of the illumination, it can be controlled by the aperture of the condenser. Here, we use indifferently both, BF and PhC images, although images from other contrast modes, such as dark-field, differential interference contrast (DIC) and cross-polarized microscopy are intrinsically different to typical halo-contrasted images, they could be also analyzed by introducing changes in the parameters that determine the definition of the contrast profile in the proposed algorithm.

In this work, microscopy images are obtained with an inverted microscopy (Nikon Eclipse 80i), which is equipped with high-performance objectives with all possible aberrations, included chromatic, sufficiently minimized. The BF mode is implemented at wide-field illumination with white light from a mercury lamp, and an apochromatic bright-field objective (x100, 1.45 N.A.). The PhC mode is implemented with a condenser equipped with an annular phase plate that focusses the illuminating light from a cold-light LED source (150mW max) in the sample, and an apochromatic phase-contrast objective (x100, 1.45 N.A.). The optical system is equipped with auto-focus, which allows for automatic correction of eventual image defocusing once the equatorial-plane of the contour object has been defined. The microscopy images are continuously recorded with high-dynamical range digital camera (Photron FASTCAM SA-3). This camera has a CMOS sensor allowing for very high sample rates through the parallel and independent read-out of each individual pixel. Each pixel possesses separated electronics for converting the accumulated charge in a pixel into a digital signal. As a result, each pixel possesses different electronic characteristics regarding its read-out noise and dark-current. The whole setup is mechanically isolated by placing on top of an anti-vibration table (Integrity 1VCS, Newport).

### Software: Algorithm description

#### Image preprocessing

Because a CMOS high-velocity camera with an in-parallel pixel-grid readout is used, the contrast images possess embedded a pixel-grid structure, which if not corrected, can affect the segmentation results. Furthermore, aberrations in the optical path can lead to artifacts in the image that can affect the segmentation result. To correct both artifacts we perform a background-correction of each movie frame before segmentation. For this we record and average over a large number of dark-field images *D*_*d*_ as well as a large number of empty (“flat”) frames *F*_*f*_ (usually ~1000 frames each). The dark-field images are recorded once for the given exposure-time and serve to correct the cameras dark-current. The flat-fields are recorded previous to and for each measurement (each movie), since their image intensity needs to match that of the measurement as close as possible. The corrected frame $I_{i}^{final}$ is then obtained from *I*_*i*_ by performing the following operation for each pixel:

$$I_{i}^{final}=\frac{I_{i}-{\langle D_{d}\rangle }_{d}}{\langle F_{f}-{\langle D_{d}\rangle }_{d}\rangle_{f}}$$

We perform the averaging over 〈*D*_*d*_〉_*d*_ and 〈*F*_*f*_〉_*f*_ in order to minimize any noise introduced when performing the correction above.

#### Contour determination

**Coordinates.** The segmentation algorithm uses both Cartesian ***P***_*i*_ and polar (*ϕ*_*i*_,*R*_*i*_) coordinates relative to the contour center ***O*** to represent the *i* = 0…*N* − 1 contour coordinates (see Table 1 for parameter values) that define the *N* contour sites. Additionally, it uses the contour normal ***n***_*i*_ which is calculated from the halo gradient at every contour site *i*. To perform its work, the algorithm uses the $P_{i}^{\prime }$, ***n***′_*i*_, and ***O***′ of the previous iteration (indicated by the prime) and uses them as the starting points to determine the ***P***_*i*_, ***n***_*i*_ and ***O*** in the following iteration. This allows the positions contour sites *i* to be refined independently of each other in an iterative manner, as we will describe below. Initial segmentation of the first image is performed sequentially, since no previous coordinates $P_{i}^{\prime }$ are available. It is initiated by providing an approximate starting point ***P***′_***i* = 0**_ on the halo and an approximate contour center ***O***′. The point ***P***′_***i* = 0**_ is then refined once to obtain the refined coordinate ***P***_***i* = 0**_ or (*ϕ*_*i* = 0_,*R*_*i* = 0_). The radius *R*_*i*_ is then used as the starting point (*ϕ*_i+1_,*R*_*i*_) to calculate the following coordinate ***P***′_***i* = 1**_, which then is again refined to obtain ***P***_***i* = 1**_. This is then continued for all *N* contour angles, which are fixed to the values *ϕ*_*i*_ = (2*π*/*N*) × *i*. After initial segmentation is completed, the algorithm writes the obtained coordinate set ***P***_***i***_ and ***O*** to $P_{i}^{\prime }$ and ***O***′ to use them as the input for the following segmentation iteration. From then on, all contour sites *i* are determined independently of each other by using the $P_{i}^{\prime },$ ***n***′_*i*_ and ***O***′ of the previous iteration, even when switching from on video image to the next.

**Table 1:** Parameter values used for imaging and tracking RBCs.

*N*	Φ	*M*	*f*_interp_	*n*_fit_
2048	π/4	64	20 px^-1^	100
*n*_bkgr_	*L*_*Y*_	Δ_shift_	Δ_coord_	Δ_center_
400	400	10 px	0.01 px	0.01 px

Parameter values used for imaging and tracking RBCs to obtain the results presented in this paper.

**Pixel grid.** The pixel size determines the maximal resolution for sampling image intensities from phase contrast halo. Without interpolation of image pixels, the processing of image intensities by an algorithm will therefore be confined to the location of pixels within the pixel grid. In particular, fitting of the halos intensity is then confined to the center position of individual pixels and can only be performed parallel and diagonal to the pixel-grid. However, for any closed contour the maximal gradient of the halo will point into every possible angular direction of the image, including direction not commensurate with those of the pixel grid. Without interpolation, the rectangular geometry of the pixel grid therefore represents a significant limitation to algorithms that are based on intensity gradients or the fitting of pixel intensities. Pécréaux and cols. handled this limitation by calculating *local* contour coordinates along three of the four pixel-grid directions, while excluding the direction closest to the contours tangent (see Fig 7 in ref. [13]). Each *local* contour coordinate is obtained by fitting a line to the halo intensity and calculating its intercept point with the background intensity at the outside contour region, which is calculated as an averaged value along the radial direction. To minimize mapping errors due to optical heterogeneities, only single cells that remain isolated from each other are considered. The final contour coordinate at a given pixel position is then defined by the weighted average of these *local* contour coordinate, where each direction is weighted by the corresponding slope of the intensity gradient in that direction. Consequently, the direction that is closest to the contour normal will be counted most since in that direction the slope will be the steepest. In our algorithm, we incorporate the concept of using weighted averages of different directions but use a more sophisticated sampling technique to find the direction and amplitude of the maximal normal gradient.

**Fig 2:**
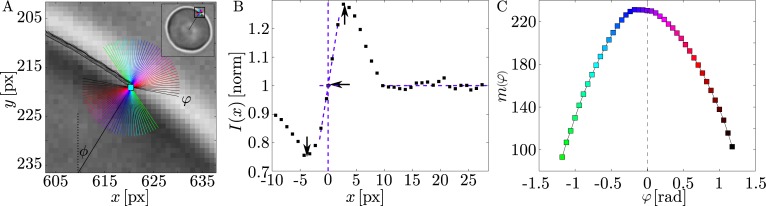
The new algorithm uses oversampling of the intensity gradient to determine the membrane position with sub-pixel precision. (A) Illustration of the local angles *φ*_*j*_ and their position on the halo. (B) Determination of local coordinate ***p***_*j*_ in a given direction *φ*_*j*_. The fit is performed between the intensity minimum and maximum indicated by the arrows. This corresponds to **Method 1** explained in the text. Note that the fit position (horizontal arrow) lies in-between the positions and intensity values of the individual pixels (black squares). This is made possible by the linear interpolation. The background intensity is determined just outside the halo. (C) Fit slopes as a function of the local angle. The maximum is perpendicular to the halo.

**Image sampling and gradient fits.** By interpolating and resampling the pixel intensities of the image during segmentation, we are neither limited to the discrete pixel positions nor the diagonal and parallel directions of the pixel grid. Instead, using the contour coordinate $P_{i}^{\prime }$ from the previous segmentation iteration as the base-point, we determine a local contour positions ***p***_***j***_ for a range of angles *φ*_*j*_ (*j* = 0…*M* − 1) (see Fig 2A) by fitting a line to the halo intensity and calculating its intercept with the background intensity similarly to Pécréaux’ method. The angle range Φ of *φ*_*j*_ is centered on the contour normal ***n***′_***j***_ of the previous iteration (see Eq (2)). The intensity values that are used for the calculations are obtained by interpolating the pixel intensities along a line in each direction *φ*_*j*_.

The gradient fit in the direction *φ*_*j*_ is determined by performing a linear fit to the intensity using the equation *y*_*j*_ = *m*_*j*_ *x* + *b*_*j*_. By using an oversampling factor *f*_interp_ (see Table 1), we are able to finely adjust the location along the intensity profile at which the linear fit is performed. As we will see in the analysis section, this is important to avoid pixel artifacts. The background intensity 〈*I*_*j*_〉 is calculated from a line in the direction of *φ*_*j*_ just outside the halo of the phase contrast image by averaging over *n*_bkgr_ interpolated intensity values (see Fig 2B). We determine the background intensity in this manner and not centered about $P_{i}^{\prime }$ (like Pécréaux does), because many cells, including RBCs, cause substantial light absorption and other image inhomogeneities, so that the light intensities inside are modified.

**Contour vector-coordinates**. The local contour positions ***p***_***j***_ in the direction of each angle *φ*_*j*_ are then given by the intercept of the fitted straight with the background intensity

$$x_{j}=\frac{\langle I_{j}\rangle -b_{j}}{m_{j}}$$

using the expressions

$$p_{j}={P\prime }_{i}+x_{j}e_{j},$$

where ***e***_*j*_ is the unit vector in the direction of the local angle *φ*_*j*_. Finally, the updated contour coordinate ***P***_*i*_ in the direction of *ϕ*_*i*_ is determined by calculating the weighted mean of the ***p***_*j*_ using their corresponding inclines *m*_*j*_ as weights:

$$P_{i}=\frac{\sum_{j}p_{j}m_{j}}{\sum_{j}m_{j}}$$ (1)

The updated contour normal ***n***_*i*_ is calculated similarly through the weighted mean of the unit vectors ***e***_*j*_ weighted by their corresponding gradient slopes *m*_*j*_ (indicated as black arrows in Fig 2A):

$$n_{i}=\frac{\sum_{j}e_{j}m_{j}}{\left|\sum_{j}e_{j}m_{j}\right|}$$ (2)

Since we perform this operation during various iterations for each video image, the algorithm iteratively finds the optimal contour position ***P***_*i*_ and normal ***n***_*i*_, about which the final directions *φ*_*j*_, at which we sample, are distributed symmetrically.

#### Coordinate sanity check

After each segmentation iteration, the algorithm checks the contour coordinates ***P***_*i*_ for any spurious outliers that could have been tracked incorrectly. This is important as the contour center in each iteration is determined as the weighted sum of ***P***_*i*_, which is weighted by the distance *ds*_*i*_ between them (see below). If a contour coordinate ***P***_*i*_ is a strong outlier and far from its neighboring coordinates ***P***_*i*−1_ and ***P***_*i*+1_, this can cause the center to jump and potentially lead to the failure of the algorithm. To avoid this, each coordinate ***P***_*i*_ from the current iteration is compared to its value of the previous iteration ***P***′_*i*_. If the distance between the two is larger than the tolerance Δ_shift_, then the value of ***P***_*i*_ and the contour normal ***n***_*i*_ is replaced with the linear interpolation of its closest neighboring coordinates that were tracked correctly, so that:

$$if:\;|P_{i}-{P\prime }_{i}|>\Delta_{shift}$$

$$then\;set:\;P_{i}=\frac{P_{i-\alpha }-P_{i+\beta }}{\alpha +\beta }$$

and calculate the normal vector as:

$$n_{i}=\frac{n_{i-\alpha }+n_{i+\beta }}{\alpha +\beta }/\left|\frac{n_{i-\alpha }+n_{i+\beta }}{\alpha +\beta }\right|$$

Here *i* − *α* and *i* + *β* are the indices of the closest neighboring coordinates that were tracked correctly. Note, that the value of Δ_coord–shift_ needs to be chosen as function of the frequency at which the video is recorded, since for lower frequencies the movement of an object contour will likely be larger between consecutive movie frames.

#### Calculation of center coordinate

The updated contour center ***O*** for the following segmentation iteration is calculated after the coordinates were checked and possibly corrected. Following Pécréaux the center is calculated as the weighted mean of the contour coordinates ***P***_*i*_

$$O=\sum_{i=0}^{N-1}P_{i}\frac{ds_{i}+ds_{i+1}}{2}$$

where the weighting factors *ds*_*i*_ are the distances between adjacent contour points:

$$ds_{i}=|P_{i}-P_{i-1}|=\sqrt{{\left(x_{i}-x_{i-1}\right)}^{2}+{\left(y_{i}-y_{i-1}\right)}^{2}}$$

Calculating the contour center in this way, we weight coordinates that are farther apart (e.g. for fixed contour-angles farther from the center) more strongly than closely spaced coordinates. This makes the algorithm converge more rapidly to the optimal center of the contour than the barycenter of the points ***P***_*i*_ would.

#### Coordinate interpolation

Because we determine the contour coordinates ***P***_*i*_ through the weighted mean of the local coordinates ***p***_***j***_, the contour coordinates ***P***_*i*_ could start to “wander” away from their originally intended location at the desired angle *ϕ*_*i*_. This can happen especially when, either the weighting factors *m*_*j*_ or the local coordinates ***p***_*j*_, are unevenly distributed tangentially to the contour. To correct this undesirable effect, the contour coordinates are updated after each segmentation iteration, using linear interpolation, to determine their position at the desired angle *ϕ*_*i*_. Briefly, we first denote the coordinate value of ***P***_*i*_ directly after averaging over its local coordinates ***p***_*j*_ with ${\tilde{P}}_{i}$ at contour angle ${\tilde{\phi }}_{i}$; then, we determine the intercept between the straight $y_{i}=\alpha e_{\phi_{i}}+O$ and the contour-segment ${\tilde{P}}_{i-1}{\tilde{P}}_{i}$ through which it passes, where $e_{\phi_{i}}$ is the unit-vector in the direction of the desired angle *ϕ*_*i*_ and ***O*** is the updated contour center. Finally, this result yields the final position ***P***_***i***_. To determine the line-segment ${\tilde{P}}_{i-1}{\tilde{P}}_{i}$ through which ***y***_*i*_ passes, we determine the two adjacent contour coordinates ${\tilde{P}}_{i-1}$ and ${\tilde{P}}_{i}$ with corresponding angles ${\tilde{\phi }}_{i-1}$ and ${\tilde{\phi }}_{i}$, so that ${\tilde{\phi }}_{i-1}<\phi_{i}\leq {\tilde{\phi }}_{i}$. The contour coordinates are then updated with the interpolated coordinates and written to ***P*′**_*i*_ as input for the following iteration. Note that we do not interpolate the value of ***n***_*i*_, since we hope the contour normal to converge to its correct value by itself over the course of the iterations. Furthermore, the linear interpolation does not represent a loss in precision, since it only pins the position of each coordinate to its desired contour angle *ϕ*_*i*_, but we still let the coordinate converge to its optimal position at that angle.

#### Segmentation termination

To determine when to terminate the segmentation process of an image, the algorithm uses two conditions for the contour coordinates ***P***_*i*_ and the contour center ***O***. After each iteration it checks whether the positions of the ***P***_*i*_ and ***O*** have changed more than a user-defined tolerance Δ_coord_ and Δ_*center*_ respectively. Thus, segmentation of an image is finished, when the following conditions are met:

$$\left|O-O\prime \right|<\Delta_{\mathrm{center}}\;\mathrm{and}\;|P_{i}-P\prime_{i}|<\Delta_{\mathrm{coord}}\;\forall \;i\epsilon N,$$

where again ***P***′_*i*_ and ***O***′ are the corresponding values from the previous iteration. Furthermore, the algorithm will terminate the segmentation process of an image after a user-defined, maximum number of segmentation iterations *N*_*max*_, if the convergence condition was not achieved previously. Note that Δ_coord_ does not correspond to the segmentation precision *δ*_1*σ*_ of the algorithm, which is discussed further below. Δ_coord_ usually has a significantly lower value than *δ*_1*σ*_, since it is the convergence criterion for an individual image only, while *δ*_1*σ*_ is the result of coordinate fluctuations between images caused by their finite SNR. In case after maximal iterations the convergence criterion is not raised with a given image, the algorithm will restart the segmentation with the next image using the contour coordinates obtained in the former, although not converged. In practice, this does not pose a big problem, since only very few images (0 up to 10, maximum, among several tens thousands of frames in a movie) do not converge. If the criterion is continually not met, when the tolerance is increased, then the image material is considered o insufficient quality.

#### Refinement of linear fit position

To avoid artifacts that can arise from performing the linear fit at a location away from where the gradient is the largest, the algorithm determines for each local direction *φ*_*j*_ at each ***P***_*i*_ the position at which *m*_*j*_ is maximal. Two methods were conceived for doing this:

**Method 1** For each direction *φ*_*j*_ we determine the location of the intensity minimum *I*_min_ and maximum *I*_max_ of the halo and situates the center position for the linear fit at the location in-between the two extremes, where the intensity value is similar to *I*_center_ = *I*_min_ + (*I*_max_ − *I*_min_)/2. On the programming level this is done by finding the maximum and minimum values in the array *Y*_*j*_ holding the intensity values of each direction *φ*_*j*_ and then determining the index *ι*_cen_ of the intensity value that comes closes to the value of *I*_center_. The linear fit is then performed over the index-range [*ι*_cen_ − *f*_interp_ ∙ *n*_fit_/2,*ι*_cen_ + *f*_interp_ ∙ *n*_fit_/2] of *Y*_*j*_, where *n*_fit_ is the length over which the linear fit is done (in [px]) and *f*_interp_ is the number of how many points will be interpolated for each pixel.

**Method 2** In this case the algorithm performs a direct search on *Y*_*j*_ for the array-index *ι*_cen_ at which the incline is maximal. It does this by performing linear fits centered at array-indexes *ι* over the range [*L*_*Y*_ − *f*_interp_ ∙ *N*_*ι*_/2,*L*_*Y*_ + *f*_interp_ ∙ *N*_*ι*_/2], where *L*_*Y*_ is the length of the array *Y*_*j*_ containing the intensity values (*L*_*Y*_ is the same for all *φ*_*j*_), *N*_*ι*_ is the user-defined search range and again *f*_interp_ is the integer of how many points will be interpolated for each pixel. This refined search is performed for each local direction *φ*_*j*_.

Combined with the various segmentation iterations performed for each movie frame, both methods converge to the position where the intensity gradient is maximal. For images of quasi-circular cell contours, both of the described methods were found to give very similar segmentation performance for the final algorithm and will therefore not be treated separately in the following discussion. However, the second method has a significant advantage in cases, where the halo of the optical image is not pronounced, such as bright-field image recordings of RBC ghosts or of giant unilamellar vesicles (GUVs), which both exhibit poor contrast. In these cases, the first method can fail to correctly determine the position of the *I*_min_ and *I*_max_, rendering the algorithm unusable. The second method is much more robust in these cases. A trade-off, however, is that it is more compute intensive due to the much higher number of linear fits being performed. This results in longer segmentation times. For dense cells, such as RBCs, which provide phase contrast images with high contrast, the first method is therefore usually preferred whose segmentation performance will be analyzed in the following sections.

#### Implementation on GPU

**Fig 3:**
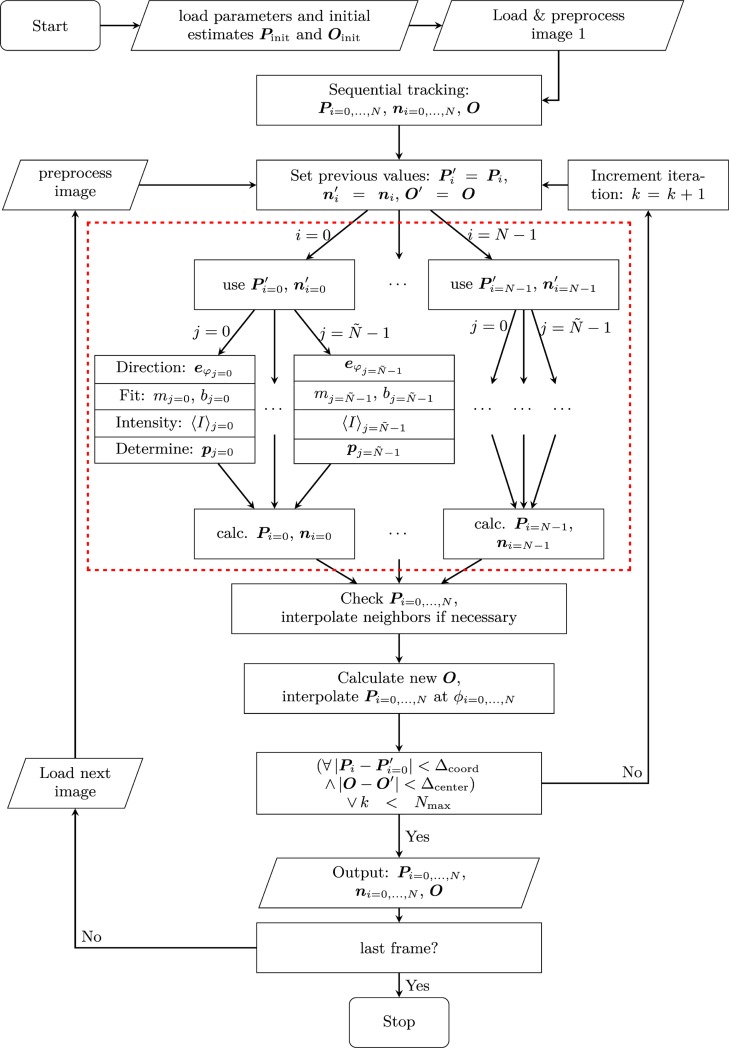
Flow-chart of the segmentation algorithm illustrating the steps that are taken in processing the movie. The section indicated by the red rectangle indicates the embarrassingly parallel part of the algorithm, which can easily be adapted for parallel processing on a GPU.

The described algorithm was designed to be parallelizable in order to be run on consumer general purpose graphics processing units (GPGPUs). All operation for the local angles *φ*_*j*_, such as the pixel interpolations, linear fits and intercept calculations are run in parallel for all contour coordinates ***P***_*i*_, so that all values ***p***_*j*,*i*_, ***e***_*j*,*i*_ and *m*_*j*,*i*_ are determined independently of each other during each iteration. The obtained results are then reduced to obtain ***P***_*i*_ and ***n***_*i*_ using Eqs (1) and (2). A flow chart of the algorithm is shown in Fig 3, where the region inside the dashed rectangle represents the highly parallelizable section. This design makes the algorithm ideally suited for acceleration on GPUs. Furthermore, since GPUs are specially designed to handle graphics, they possess dedicated hardware units for performing the bi-linear interpolation of the pixel intensities, which greatly accelerates this operation.

The algorithm was implemented using the Open Compute Language (OpenCL), making the code portable and executable on GPUs from different vendors and also conventional CPUs. Comparing the execution time of our implementation on an AMD Radeon HD 7970 to that of an Intel Xeon 5760, we registered a 100 × speed-up on GPU with respect to the CPU version of the algorithm, when imaging RBCs using the segmentation parameters listed in Table 1. Our implementation of this high-precision flicker spectroscopy contour tracking algorithm (HiPFSTA) has been made publicly available at: www.github.com/michaelmell/hipfsta

Analysis of an individual image with five segmentation iterations takes roughly 0.1s. It depends on the number of iterations per image, which for RBCs is usually 3 to 5 iterations using the listed parameter values. Therefore, execution on the GPU enables the processing of movies recorded with the FASTCAM SA-3 in a reasonable time of ~2.2hours (for 8 ∙ 10^4^ movie frames of a 40s video recorded at 2000FPS). On the CPU mentioned above this would not be feasible.

## Analysis of segmentation performance

### Tracked object examples

**Fig 4:**
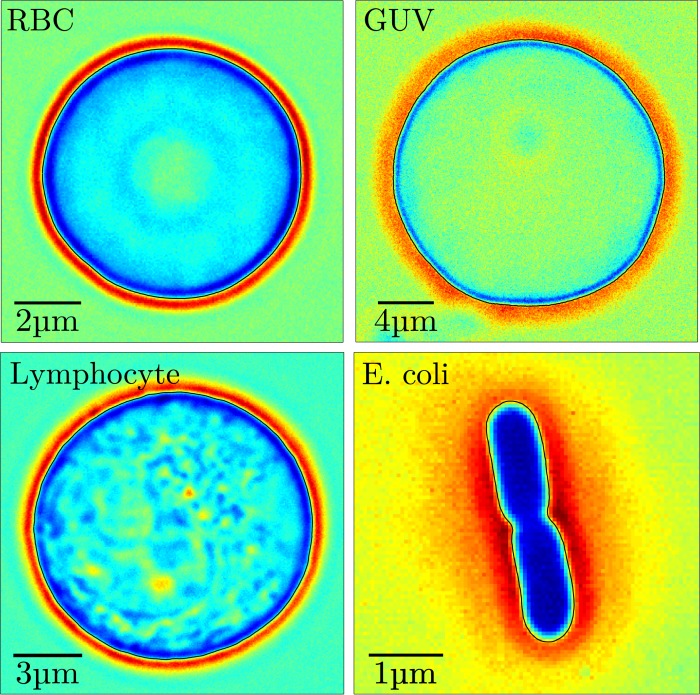
Examples of microscopic objects that have successfully been tracked using our new segmentation algorithm. The obtained contour is indicated by the black line that is overlaid on each image. All images were recorded with bright-field light-microscopy, except for the E. coli, which was recorded using a phase-contrast objective (100x, NA1.45).

In its current form, the algorithm can be applied to spherical and non-spherical objects, which fulfill two requirements: 1) They exhibit a sufficiently strong contrast halo necessary for the algorithm to function correctly. 2) It is possible to uniquely assign an individual radius value *R*_*i*_ to a corresponding angle *ϕ*_*i*_, when describing the contour in polar coordinates. The second requirement is the result of the interpolation used to fix the angular position *ϕ*_*i*_ of the contour coordinates and could be relieved by conceiving a different method. The segmentation algorithm has so far been successfully applied to various objects including RBCs, *E*. *coli*, GUVs and lymphocytes for which we show example images overlaid with their tracked contour in Fig 4. All images where obtained using bright-field microscope setup except for the image of *E*. *coli*, which was obtained using a phase-contrast objective. As we will see in the next section, the segmentation precision crucially depends on the optical contrast afforded by the object.

### Real-space

**Fig 5:**
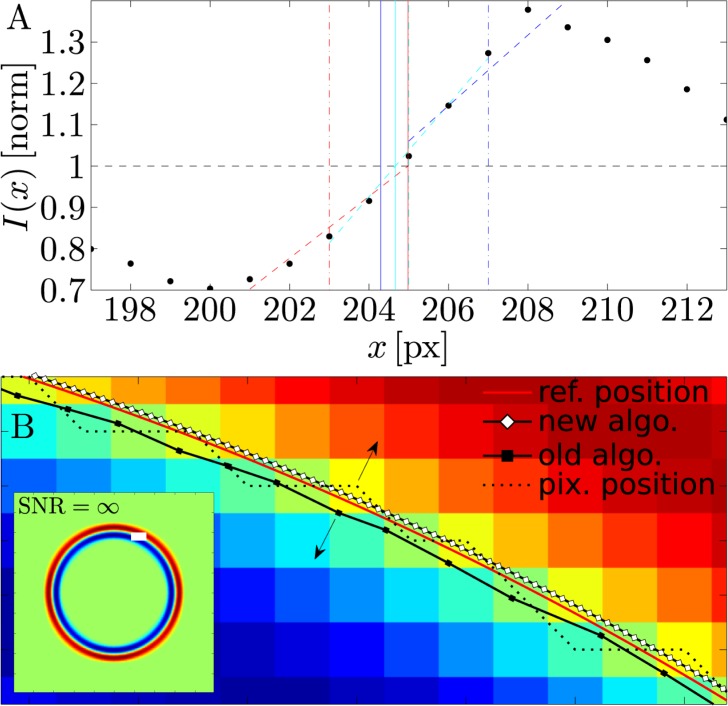
Image pixilation causes jitter in the positions obtained with the classical algorithm. (A) Illustration of how fitting at an off*s*et from the gradient maximum affects the obtained fit-slope and results in a deviation in the final coordinate. (B) Comparison of the reference contour and the contour coordinates obtained from the previous method [13], and our refined algorithm here described. The artifact described in (A) leads to transverse shifts in the final position (black squares), whenever the pixel-position about which the fit is performed (dashed line) shifts transversely to the contour. The red line represents reference position of the circle used in the synthesis of the image with SNR = ∞, which is shown in the inset indicating the shown area with a white rectangle. The tracked coordinates obtained from the new algorithm (white diamonds) follow the reference position precisely.

Our segmentation algorithm has been developed to overcome the shortcomings detected in the previous algorithm proposed in Ref. [13], for which we have found that the discretization of the pixel-grid leads to artifacts in the final contour coordinates in the algorithm. The reason for those artifacts is that the initial position for performing the linear fit is confined to the coordinates of the pixel-grid, *i*.*e*. the pixel centers, which determine the coordinates of every point in the halo. When the position of the halo moves against the pixel-grid, this will gradually change the position at which the linear fit is performed and therefore affect the incline *m* of the fitted line as the fit-position draws closer to the intensity minimum or maximum of the halo. This is illustrated in Fig 5A, where we show three fits at different positions of the intensity profile of the halo. Fitting close to the maximum or minimum alters the incline and consequently the final, tracked contour coordinate. While in reality fitting should not occur this close to the extremes, this effect is still appreciable. Fig 5B shows the effect of this on the tracked contour of a *synthetic* phase-contrast image of a smooth, structureless circle, where the solid red line represents the position of the phase boundary of the circle used for the image synthesis (see Methods). For illustration purposes, this synthetic dataset does not contain any noise and thus has an infinity signal-to-noise ratio (SNR = ∞; see Methods). The dashed black line and the black squares show the pixel position used for fitting in Pécréaux’ algorithm and the tracked contour coordinates. The white diamonds represent the tracked contour coordinates from our algorithm. As can be seen, the contour coordinates calculated from that algorithm move slightly in the opposite direction, when “jumps” in the pixel positions occur. This occurs whenever the pixel position, about which the linear fit is performed, “jumps” transversely to the halo and thus produces a slight change in the incline of the fitted straight *m* as illustrated in Fig 5A. Thus, although the image does not contain any noise, the algorithm itself causes artifacts in the final contour.

**Fig 6:**
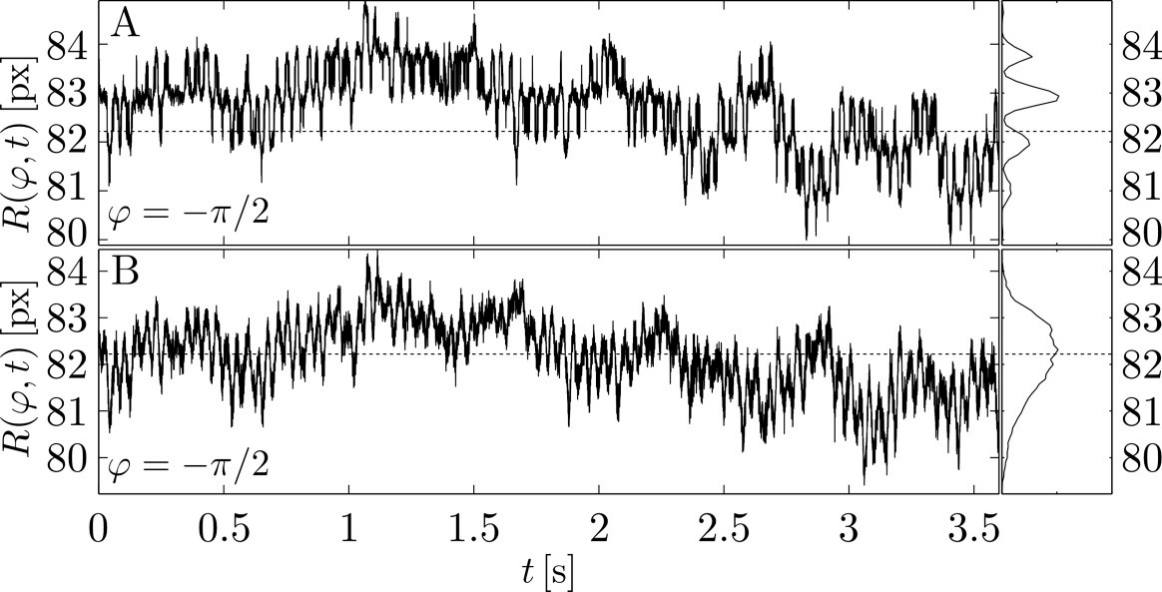
Comparison of the radius fluctuation time-series obtained from a measurement of a POPC GUV using Pécréaux’ algorithm (A) and our algorithm (B). The artifacts from imprecise fitting leads to various maxima in the displacement-distributions spaced 1px apart. This artifact is not present when using our new method.

Although this effect is subtle, it can lead to misinterpretations when analyzing the contour coordinates in real-space. This is evident when comparing time series of the contour radii *R*(*ϕ*_*i*_,*t*_*n*_) at a fixed angle *ϕ*_*i*_ as produced either by the algorithm in Ref. [13] or by our algorithm for an artificial lipid vesicle. The distribution of *R*(*ϕ*_*i*_,*t*_*n*_) from the former algorithm exhibits maxima and minima, which are spaced approximately 1px apart as shown in Fig 6A. This behavior is the direct consequence of the artifact due to pixelation described above (with pixelation we refer to the discretization of the continuous image into the discrete pixel grid). It is the reason we take great care to optimize the fit-location in our algorithm (see **Refinement of linear fit position**) and is not observed in the radius time series obtained from the same dataset using the new segmentation algorithm shown in Fig 6B.

**Fig 7:**
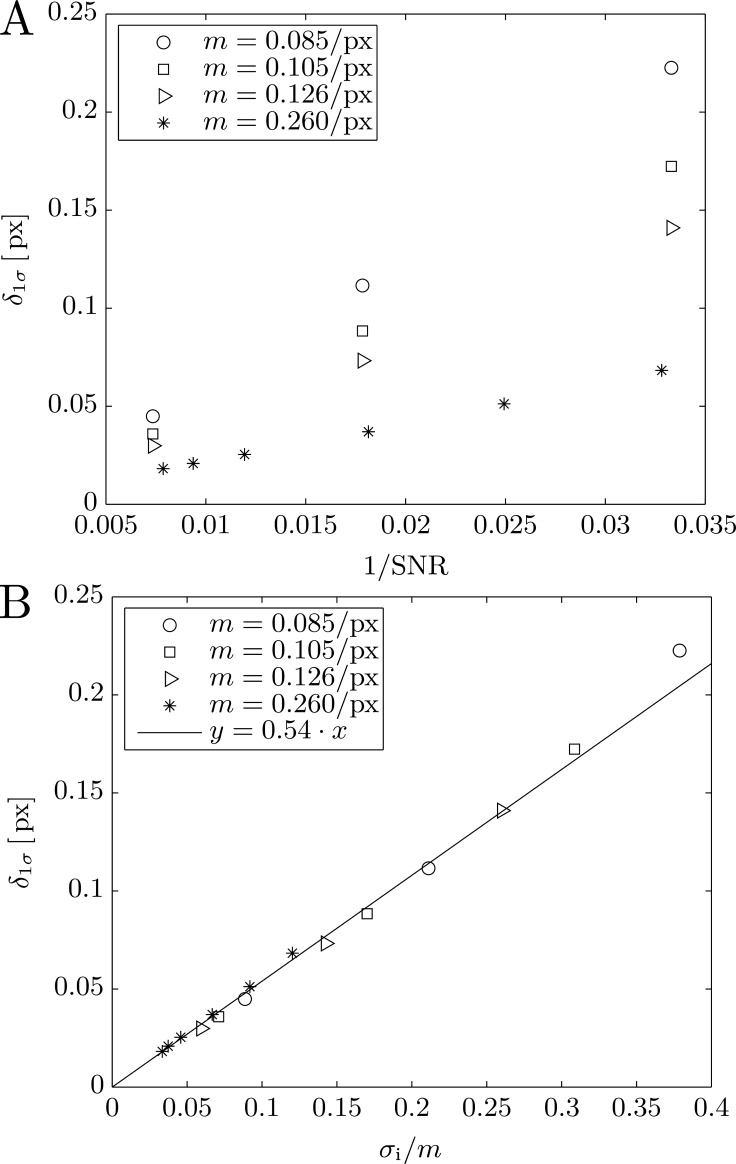
Segmentation precision depends on image noise and the slope of the intensity gradient. (A) Segmentation precision *δ*_1*σ*_ versus 1/*SNR* for a dried RBC at different exposure times (‘_*_’) and for three synthetic datasets (‘□,○ and Δ’) with different level of contrasts/slopes *m*. The precision values from each dataset fall onto a straight determined by the contrast/slope *m*. (B) After renormalizing x-axis to *σ*_*i*_/*m* all values fall onto a straight *y* = 0.54 ∙ *x* allowing for easy estimation of the segmentation precision from the two known quantities *σ*_*i*_ and *m*. Note that the contrast/slope values are for to unit background intensity.

In order to quantify the precision of the new algorithm experimentally, we recorded and tracked movies of dried RBCs, which are used as static reference objects. The dried RBC are well suited as reference fluctuation-less objects, since they exhibit a similar level of contrast and halo-shape as living RBCs. Additionally we synthesized computer-generated phase contrast images as proposed by Usenik[21]. To determine the segmentation precision, we measure the variability of the contour coordinates obtained with our algorithm. For this, we calculate the distance *δ*_*i*,*n*_ between corresponding coordinates at the same contour angle *ϕ*_*i*_ from consecutive frames *n* − 1 and *n*, so that *δ*_*i*,*n*_ = |***P***_***i*,*n***_ − ***P***_***i*,*n*−1**_|. Since the contours in the recorded image of dried RBC and the synthetic images are static, this gives us information on how much the contour position varies between frames due to the finite precision of the algorithm. We then calculate the distribution *P* of the inter-coordinate distances *δ*_*i*,*n*_ and define its standard-deviation 1*σ* as the segmentation precision *δ*_1*σ*_. Note that since *δ*_1*σ*_ is obtained from the differences of coordinates pairs its value is expected to be $\sqrt{2}\times$ larger than the error of the individual coordinates. The reason we determine the segmentation precision in this manner and not using the radius fluctuation 〈*h*(*t*)^2^〉 = 〈(*R*(*t*) − 〈*R*(*t*)〉)^2^〉 over the whole time-series, is because our method will not be affected by slight shifts in focus or vibrations of the experimental setup, which can occur on time-scales of various seconds. The positional accuracy can be estimated theoretically by determining the error in the x-position *σ*_*x*_ of the intercept between the linear regression of the intensity gradient with slope *m* and the average image intensity 〈*I*〉. This straight forward calculation yields (see SM):

$$\sigma_{x}=\frac{\sigma_{I}}{m}\sqrt{\frac{1}{U}+\frac{1}{T}}$$ (3)

with standard deviation *σ*_*I*_ of the image intensity and the slope *m* of the intensity halo. The proportionality constant $\sqrt{1/U+1/T}$ depends on the number of fitted values *U* and averaged intensity values *T*. Note that *σ*_*x*_ does not correspond directly to *δ*_1*σ*_. As mentioned above *δ*_1*σ*_ is $\sqrt{2}\times$ larger than the coordinate error, which in turn are the weighted average of *M* fits in the directions of the local angles *φ*_*j*_, each of which possess a different error *σ*_*x*_ due to different slopes *m* (see Fig 2(C)). However, the proportionality *δ*_1*σ*_ ∝ *σ*_*I*_/*m* should remain unchanged, since the directions with largest slope *m* will dominated the weighted sum. To test the theoretical estimate, we determine the segmentation precision for images with different signal-to-noise ratios (SNR) and contrast levels (different *m*) using synthetic images (see Methods). The segmentation precision *δ*_1*σ*_ is inversely proportional to the image SNR, *δ*_1*σ*_ ∝ 1/SNR as can be seen in Fig 7A. After renormalizing the abscissa to *σ*_*I*_/*m* all data points fall onto single line confirming *δ*_1*σ*_ = *σ*_*I*_/*m* ∙ *K* with proportionality constant *K*. We can compare the value of *K* to that of $\sqrt{1/U+1/T}$ to obtain an estimate of the precision improvement due to the weighted averages (see Eq (1)) compared to a single fit. Although each fit consists of *n*_fit_ data-points (see Table 1) the number of *independent* pixel values being fit is actually *U* = *n*_fit_/*f*_interp_ = 5. Accordingly, *T* = *n*_bkgr_/*f*_interp_ = 20 for the number of *independent* pixel values being averaged for the background intensity. We therefore have $\sqrt{1/U+1/T}=0.5$. From Fig 7B, we obtain the proportionality constant *K* = 0.54. Taking into account the factor $\sqrt{2}$ from calculating *δ*_1*σ*_, we estimate an improvement of $0.5/(0.54/\sqrt{2})=1.31$.

From Eq (3) it follows that we can improve segmentation precision by increasing the SNR of the image through higher illumination intensity or longer exposure times. Furthermore, the amount of contrast produced at the object boundary plays a crucial role in determining how precise the algorithm is able to track its contour, since it determines *m*. Moreover, since *m* and *σ*_*I*_ are easily obtained from a single image, this allows for simple estimation of the expected segmentation precision. Table 2 lists the data shown in Fig 7. As indicated, we achieve a positional accuracy *δ*_1*σ*_ of 0.01px to 0.03px for SNR-values of 127 to 84 corresponding to typical experimental frame rates of 1500 to 3000fps. For our setup with an effective pixel-size of 50nm this translates into a positional accuracy of below 0.9nm to 1.25nm for dried RBCs specimens. We also estimated the precision for RBCs and GUVs in buffer, which exhibit a lower contrast and therefore lower value for *m*. We find 0.05px and 0.3px respectively using values for *m* taken from bright field microscopy observations. For completeness we also include the value of $\delta_{1\sigma }/\sqrt{2}$, which is expected to represent the error an individual coordinate, instead of their difference. For a RBC in buffer, we estimate a value of $\delta_{1\sigma }/\sqrt{2}=0.04\mathrm{px}$ corresponding to 1.85nm.

**Table 2:** Values for the dried RBC datasets compared to RBCs and GUVs in buffer solution.

	RBC_dried_	RBC_dried_	RBC_dried_	RBC_dried_	RBC_dried_	RBC_dried_	RBC_buffer_	GUV
FPS	1500	2000	3000	6000	10000	15000	2000	1500
SNR	127.3	106.8	83.9	55.2	40.1	30.4	101.5	137.9
*m*	0.2355	0.251	0.259	0.272	0.272	0.273	0.1	0.014
*σ*_I_/*m*	0.033	0.037	0.046	0.067	0.092	0.120	0.097	0.517
*δ*_1_*σ*[px]	0.018	0.021	0.025	0.037	0.051	0.068	0.052*	0.279*
*δ*_1*σ*_*/*2^½^ [px]	0.013	0.015	0.018	0.026	0.036	0.048	0.037*	0.197*

Values for the dried RBC datasets shown in Fig 7 compared to typical values for measurements of RBCs and GUVs in buffer solution. The imaging precision δ_1σ_ and δ_1σ_/2^½^for the latter two cases was extrapolated from their values for *σ*_*I*_/*m* using the equation δ_1σ_ = 0.54 σ_I_/m (indicated by *).

The segmentation precisions for GUVs previously reported by Pécréaux [13] and Usenik [21] are not exactly comparably to our results, since these authors used phase-contrast, instead of bright field microscopy. We can however attempt to estimate the precision that we would expect from our algorithm. From Eq (1) and Fig 1 we estimate an intensity-normalized *m* = ((*I*_max_ − *I*_min_)/〈I〉)/*n*_pix_ = ((525 − 435)/480)/8px, where *I*_min_ and *I*_max_ are the minimum and maximum intensity of the halo, 〈*I*〉 is the average and *n*_pix_ is the number of pixels in the gradient-range. For *σ*_*I*_ we estimate *σ*_*I*_ = 3/〈*I*〉 = 0.007, assuming a standard deviation of 3 grey level units. Using these values we obtain an estimated segmentation precision of *δ*_1*σ*_ = 0.54 ∙ *σ*/*m* = 0.16px, which is comparable to the 0.1px-precission that Pécréaux claimed for his algorithm [13]. Usenik et al. [21] claimed a precision of 26.9 to 34.1nm for their algorithm at a pixel-size of 35nm. Therefore, their precision is not significantly below pixel-size and we expect our algorithm to perform significantly better. However, the value for the latter case was achieved at SNR = 23 dB, which for our SNR-definition corresponds to a very low SNR = 10^23/20^ = 14.13 (see Methods). Finally, for RBCs, the accuracy of our new software implementable with contrast imaging is competitive with other sophisticated microscopy hardware such as the diffraction phase microscope [33] used by the authors of references [9,12,34], who report an accuracy of 3.3nm for the RBCs thickness-fluctuations due to the DPMs limited optical path stability [22,33]. However, their experimental setup uses a 40× (NA0.65) oil-immersion objective with 400nm resolution. Since we use an oil-immersion objective with 100 × magnification (NA 1.4), we should have better lateral resolution along the contour, which should translate into a diffraction-limited lateral resolution of around 200nm. The proposed method deals with the analysis of the contrast halo, which has been implemented as a super-localization software that allows contour segmentation at sub-pixel resolution with bright field/phase contrast images. However, our super-localization method can be considered broader in scope, as potentially implementable with other optical settings no dealing with contrast halos. In dark-field, for instance, amplitude dependent glows formed at the rim of the object are potentially detectable by our software. Cells with sufficient optical contrast, such as RBCs, may be efficiently segmented using images obtained upon a high scattering luminosity. Differential interference contrast (DIC) has no halo yet, however, contour segmentation could be performed after image integration leading to sufficient membrane contrast.

#### Fourier space analysis

To analyze the performance of our algorithm in Fourier space and quantify the effect that limited segmentation accuracy has on, we calculate the spectra obtained from the synthesized datasets. Calculation of the spectra is done as described by Pécréaux [13]. Briefly, a tracked contour (*R*_*i*_,*ϕ*_*i*_) is transformed into a height-profile (*h*_*i*_,*ϕ*_*i*_) above and below a circular equilibrium radius *R*. The *h*_*i*_ are then decomposed into their Fourier components, *n*, which for the continuous case can be written as

$$h(\phi )=R\left[1+\sum_{n=1}^{\infty }a_{n}\;\cos\left(n\phi \right)+b_{n\;}\sin\left(n\phi \right)\right],$$

where *R* is the equilibrium radius of the contour. The spectrum is then given by the RMSD of the modulus $|c_{n}|=\sqrt{a_{n}^{2}+b_{n}^{2}}$. The spectrum is then given by the RMSD of the modulus $|c_{n}|=\sqrt{a_{n}^{2}+b_{n}^{2}}$. Here we use the normalization proposed by Pécréaux [13], so that absolute values are directly comparable to his work. The final spectrum is then given by

$$\langle {\left|u_{n}\right|}^{2}\rangle =\frac{\pi {\langle R\rangle }^{3}}{2}(\langle {\left|c_{n}\right|}^{2}\rangle -{\langle \left|c_{n}\right|\rangle }^{2}),$$

where the wavenumber *q* relates to the mode number *n* through *q* = *n*/*R*.

Above a given wavenumber compatible with a minimal resolvable wavelength, the segmentation algorithm in Ref. [13] produces a spectrum dominated by noise, which continues to increase towards higher wavenumbers. The authors attribute this to the discrete detection of the contour, which causes an increased floor noise as the wavelength of the mode becomes smaller than 4 × pixel-size. He therefor argues that as a result of a such discretization there exist two main limitation to his algorithm: 1) the amplitude precision orthogonal to the contour, and 2) the sampling precision in parallel to the contour [13].

**Fig 8:**
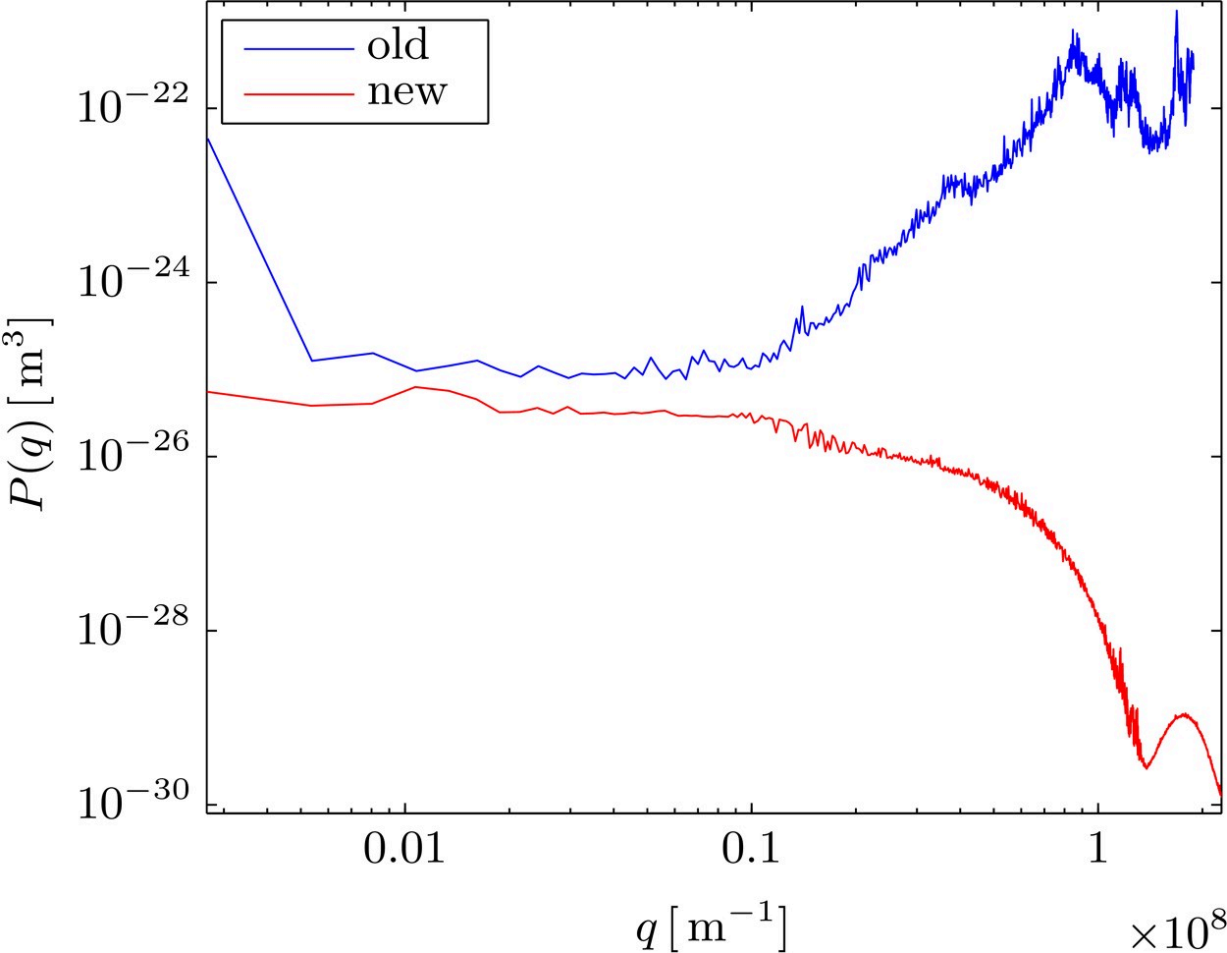
Comparison of spectra obtained from the same dataset (SNR = 106.7) using the new and old algorithm. The spectrum from the old algorithm increase at high *q*, since the pixilation artifact causes increased noise as the wavelength draws closer to the pixel-size. The spectrum from the new algorithm exhibits a minimum at the location corresponding to wavenumber *q*_pix_ of a pixel.

**Fig 9:**
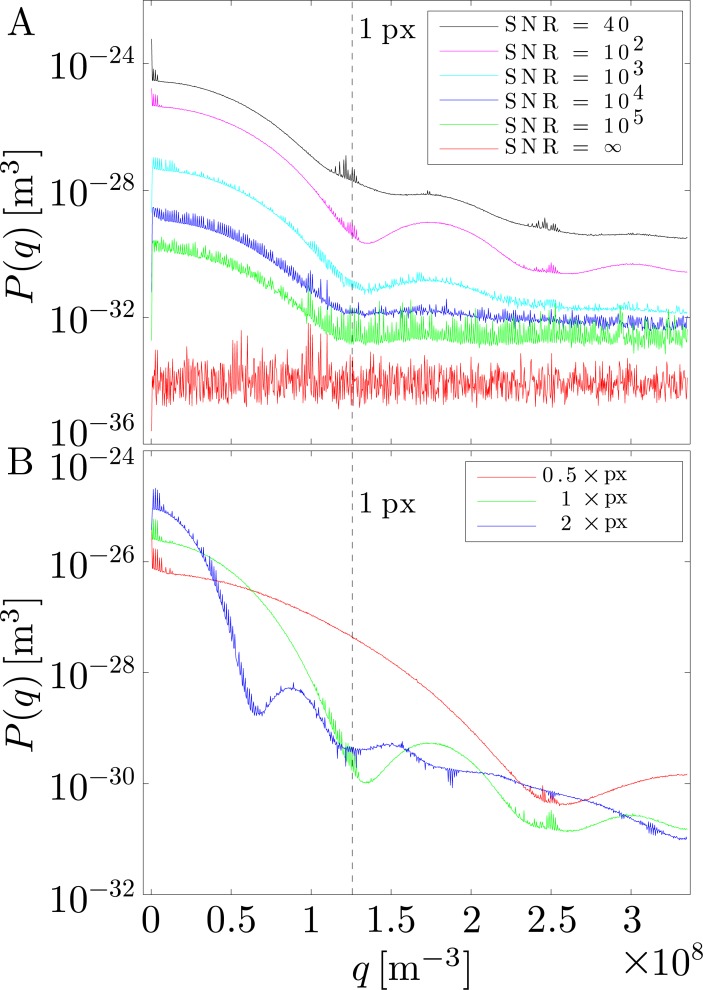
Shape and level of the noise floor depends on the pixel size and SNR. (A) Illustration of the inverse scaling behavior of the spectrum with pixel size. 1 × px corresponds to the experimental pixel size. The shape of the noise floor is the result of the pixel-noise being convoluted with kernel from the linear interpolation. (B) Plot of the spectra obtained for different SNRs. The noise floor decreases for higher (better) SNRs. At high SNRs (SNR ≥ 10^4^) it begins to enter the noise due to numeric imprecision. Since this noise is independent of pixels, spectrum for SNR = ∞ is flat (red line).

The spectra obtained for the synthetic spherical object with the new algorithm have a significantly different shape than the one obtained from Pécréaux’ algorithm (see Figs 8 and 9). Whereas our algorithm yields a spectral shape that is compatible with the pixel-grid structure, the algorithm in Ref. [13], however, yields spurious amplitudes in the whole range of wavenumbers analyzed. Indeed, our algorithm computes lower amplitudes, which are compatible with the level of noise artificially introduced for these ideal spherical objects. A representative example is shown in Fig 8 and compared to the spectrum obtained from Pécréaux’ algorithm for a synthesized dataset (SNR = 106.7). The shape of the fluctuation spectrum is reminiscent of a sinc-function, with its first minimum located roughly at the wavenumber *q*_pix_ corresponding to the size of 1px. This spectrum represents the spectral noise floor resulting from the finite SNR of the synthesized images. It is the result of the noise of the individual pixels, whose influence on the final spectrum is convoluted with the frequency response of the linear interpolation used in the segmentation algorithm. A linear interpolation can be written as a convolution of the sample values with triangular pulse [35], whose Fourier transform and thus frequency response is proportional to a sinc^2^ function. It is this dependency of the form factor of static objects, which causes the noise floor *not* to be evenly distributed across the spectrum, but to follow a sinc-like shape corresponding to the structure form factor of noisy pixels. By changing the pixel size in the synthesized images, we confirm the inverse scaling behavior expected for the pixel-structure form factor (see Fig 9A). Note that it is therefore possible to change the shape of the spectral noise floor by changing the effective pixel-size either experimentally or through binning and other forms of digital image pre-processing, such as rescaling with bi-cubic interpolation.

Our findings lead us to a slightly different conclusion of the spectral increase of Pécréaux’ algorithm at large wavenumbers. Considering the Nyquist-Shannon sampling theorem the theoretical cutoff for spatial frequencies that can still be resolved is *q*_Ny_ = *N*/(2 ∙ *R*) [36]. For Pécréaux’ algorithm with on average one contour coordinate point per pixel this should approximately be the inverse of two times the wavenumber of the image pixel-size: *q*_Ny_ ~ *q*_pix_/2. Modes with wavenumbers *q* > *q*_Ny_ should exhibit aliasing and therefor increase in spectral amplitude. This is observed for synthetic datasets using our algorithm, when *q* > *q*_Ny_ = *N*/(2 ∙ *R*) = 2048/(2 ∙ 61px) = 16.8px^−1^ (see SM). The fact that the cross-over wavenumber *q*_c_ above which noise starts dominating in Pécréaux’ algorithm is much smaller than *q*_c_ = *q*_pix_/2, suggests that the observed increase in spectral amplitude is primarily due to the artifacts in the determined orthogonal contour positions (see Figs 5 and 6). These transverse artifacts due to pixelation will increasingly affect the mode amplitudeswhen the modes’ wavenumbers become similar to *q*_pix_. The new segmentation algorithm does not produce these artifacts in the transverse coordinate, neither propagates errors along the longitudinal position in the contour, differently to the previous method, which determines coordinates in a given point from the formers. That method is thus prone to propagate possible errors to adjacent points in the contour, which favors (in Fourier space) multiplicative deviations at high wavenumbers (*q* ≥ *q*_*px*_), just in the region where it becomes inapplicable.

To better understand the effect of image noise on the resulting spectrum from our algorithm, we synthesized various datasets at different SNRs. The synthetized images correspond to ideal circular contours with a pixelation noise superposed. As expected, the noise floor decreases with increasing SNR (see Fig 9A), which can be interpreted as the form-factor of the noisy pixel-grid; this is, in Fourier space, the convolution between an instrumental white noise (the electronic noise introduced by the camera) with the linearly-interpolated square grid the contour is embedded. At very high SNRs the numerical precision of the segmentation algorithm itself becomes the limiting factor. Since it is independent of the images’ pixels, it results in a *q*-independent noise floor in the spectrum that for SNR ≥ 10^4^ increasingly dominates the high-*q* range of the spectral noise floor from pixel-noise, until it is the only noise component at SNR = ∞. This suggests that the segmentation precision of our algorithm may experimentally only be limited by the image SNR and in the future could be improved by using new low-noise, digital imaging devices such as scientific CMOS (sCMOS) cameras [37].

#### Experimental fluctuation spectra

**Fig 10:**
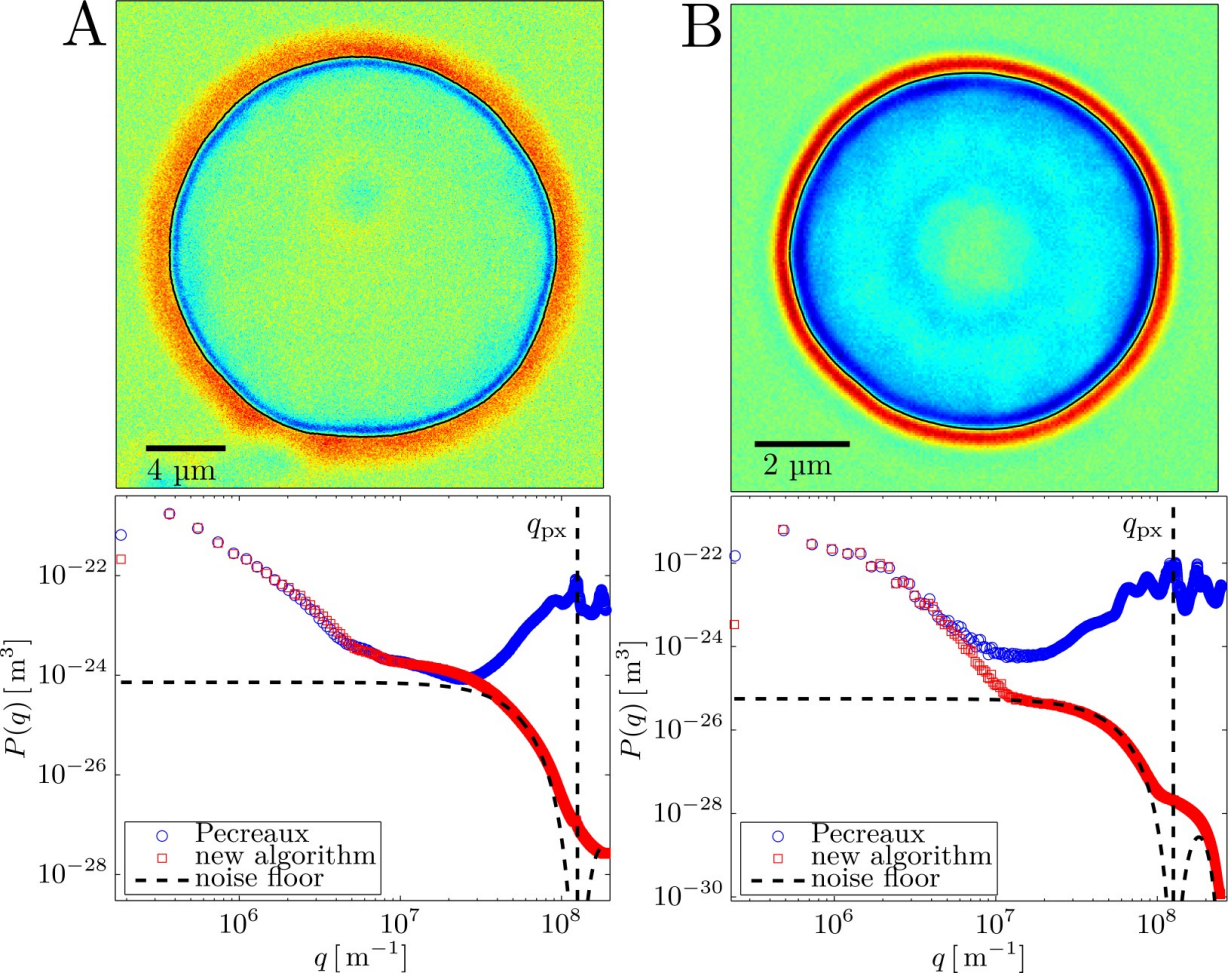
Example flicker spectra of GUV and RBC datasets obtained with the classical and new segmentation algorithms. Top panels: Instantaneous snapshot of a fluctuating GUV made of POPC (A) a healthy RBC undergoing flicker motions (B). The microscopy images are taken with a CMOS camera in the PhC mode at the equatorial plane of the quasi-spherical objects (see Methods for details), and correspond to one frame taken arbitrarily in a movie recorded at 6kfps. Lower panels: Fourier space fluctuation spectra obtained from the statistical analysis of the membrane contour fluctuations. Results from the proposed new contour-segmentation algorithm are compared with results from the classical algorithm described in [13]. The pixelation form-factor, as empirically obtained from optically equivalent synthetic contours (non-fluctuating but including similar noise than the CMOS camera), are plotted as dashed lines with the characteristic sinc-like shape of a squared pixel grid. These lines represent the minimal floor noise corresponding to the instrumental “fluctuations” due to the electronic noise of the CMOS camera implemented with the current optical configuration. The wavenumber corresponding to a distance of one-pixel in Fourier space is plotted as a vertical dotted line labelled q_px_ = 2π/px.

Although this work is almost focused in the description of the algorithmic aspects of our improved segmentation method, we will finish by discussing on its applicability to study the spectrum of the membrane fluctuations in real fluctuating objects. Fig 10 compares the results obtained from our proposed algorithm with those from the Pecréaux’ method, in two very different cases: A) Thermal fluctuations in giant unilamellar vesicles (GUVs) made of a very flexible, single lipid (POPC) bilayer membrane, and a relatively low optical contrast between the vesicle lumen and the exterior. B) Membrane fluctuations in healthy red blood cells (RBCs) with a complex lipid bilayer coupled to a rigid spectrin skeleton. Due to the presence of high amounts of hemoglobin, and other cytoplasmic proteins, the optical contrast is in this case much higher than with the former case of empty GUVs. The upper images correspond to instantaneous photographs of the near-circular fluctuating objects at the equatorial plane, and the lower plots represent the amplitude spectra of the membrane fluctuations as decomposed in Fourier space (see section “Fourier space analysis”). In the case of flexible GUVs, the amplitude of the shape fluctuations is large, which makes the spectral amplitudes to be quite high, and resolvable, at low wavenumbers corresponding to distances larger than the pixel grid (*q*_*pix*_ = 1.25 10^−7^ m^-1^). At *q* << *q*_*pix*_ both algorithms give almost identical results, which indicates the mutual robustness of the two segmentation algorithms to determine spatial correlations between membrane elements separated far beyond one pixel. However, since the optical contrast is low in this case, hence the SNR quite low, then the floor noise arising from contour pixelation is rather high, at *q* ≥ *q*_*pix*_ becoming of the same order as the fluctuation amplitudes (see Fig 10A bottom). In that high-q regime (*q* > *q*_*pix*_), the new algorithm calculates spectral amplitudes that correspond to the structure factor of the pixelized contour, which is nearly describable as a sinc-like function that can be considered the floor noise intrinsic to the considered optical configuration (see Fig 9). However, the Pecréaux’ algorithm returns in this regime spurious values of the spectral amplitudes, which arise from the sub-pixel artifacts described above. As a consequence, Pécréaux’ method is quite limited in this sub-pixel regime, where the spectral amplitudes become chiefly determined by a high pixelation noise, in this case dominated by the errors in discriminating the positions of very neighboring points in the contour. Our method, however, is very robust in determining the contour position independently of the radial direction chosen, which makes the segmentation uncertainty independent of the lateral distance between two points along the contour. In our method, consequently, the pixelation noise is just minimal, and stands at the value corresponding to the instrumental noise of the image obtained from a given optical configuration. This situation becomes especially evident with RBC images (see Fig 10B), where the higher optical contrast allows a higher SNR that translates into a much lower pixelation floor noise than in less contrasted GUV membranes. For RBCs, and other real cells, the sinc-like structure factor of the pixel grid is more than 10-fold lower than for GUVs, allowing for robust analysis of the fluctuation amplitudes at much higher wavenumbers than the previous methods. A notable advantage of the proposed algorithm, associated to its intrinsic robustness and low uncertainty in lateral segmentation, is that the floor noise due to pixelation is completely recognizable and predictable, which allows for correct identification and eventual subtraction from further deconvolution analysis. From the compared analysis above, it seems quite clear that the proposed algorithm can bring improved measurements that result in newer, and deeper, analyses than possible with former approaches.

## Conclusion

We have developed a high-precision flicker spectroscopy contour tracking algorithm based on an intensity-gradient segmentation schema (HiPFSTA, available open-source at www.github.com/michaelmell/hipfsta), which is designed to run on graphical processing units and makes extensive use of image-oversampling using the processing power of these processors. Segmentation precision was tested using images of dried RBC and synthetic images and quantified in real-space as well as Fourier space, relevant for flickering spectroscopy. The algorithm was shown to achieve segmentation precision down to 2nm for experimental images of dried RBCs at SNRs of about 100, which corresponds to frames rate of 2000fps in our experimental setup using a high-velocity CMOS camera (FASTCAM SA-3, Photron). The segmentation precision was shown to depend on the optical contrast of the observed object and the SNR of the image. Results from the spectra in Fourier space of synthetic images suggest that segmentation precision is mainly limited by the image SNR opening the possibility for future improvements to the experimental setup with newly developed, low-noise scientific CMOS (sCMOS) sensors [37].

The segmentation precision of our algorithm was found to be significantly better than that reported for the previous methods by Usenik [21] and Pécréaux [13], who reported precisions of 25 to 36nm for the position of the cell contour and an estimated 0.1px, respectively. The latter method was directly compared to our algorithm and found to exhibit significant segmentation artifacts, the source of which was concluded to be its partial dependence on the pixel grid. These artifacts were also found to be the reason for the spurious increase in spectral amplitudes in Fourier space towards high wavenumbers, which limited the observable *q*-range to the first fifty modes in the previous method [13]. This artifact, and associated properties, are not exhibited by the new algorithm, which can be practically extended up to much higher Fourier modes (*n*_*max*_ ≈ 1000), ranging in the microscopic domain (*q*_*max*_ ≈ 0.3 nm^-1^), where the fluctuation dynamics of the cytoskeleton elements can be probed over nanoscopic distances, *d* ≈ 2π/*q*_*max*_ ≈ 20 nm. The robustness of the proposed algorithm is largely superior to previous approaches to cell contour segmentation of microscopy images, in terms not only of transverse accuracy (essential to exactly determine the amplitude of the fluctuations with sub-pixel precision) but also of lateral resolution (crucial to extend spatial Fourier analysis below sub-pixel longitudinal distances). Our algorithm, and its implementation with GPU’s, has minimized the number of computational artifacts associated to any sub-pixel interpolation, so under optimal optical performance (high contrast and low camera noise), it is able to work within the instrumental limit, which determines a perfectly detectable and analyzable floor noise. The high precision of our optical microscopy method is comparable to the precision of much more sophisticated methods, such as interferometric microscopy. Our method is complementary, since their interferometric method gives a two-dimensional height map of the cell, while our method yields the membrane displacement at the equatorial plane. Finally, the spatial precision and temporal sampling rate of our method is comparable to those reported for tracking methods with optical traps. However, our method gives us access to the whole cell contour, whereas optical traps are only able to probe single points of the membrane. Finally, the new algorithm was shown to be usable for biological cells with different properties and shapes, including RBC, *E*. *coli* and lymphocytes as well as biomimetic objects such as giant unilamellar vesicles. It is expected that this method will allow for much more refined results, when applied to RBC and other mechanically compliant cells, which may yield new insights into their cell-mechanics using conventional optical contrast microscopy. The method is sufficiently robust and versatile that is potentially adaptable to other optical modes and microscopy settings.

## Materials and preparation methods

### Microscope and camera

The experimental setup consisted of a Nikon Ti2000 microscope equipped with a 100 × (NA1.4) bright-field objective followed by additional 1.5 × and 2.25 × optical zooms giving a total 337.5 × zoom. This optical system images onto a Photron FASTCAM SA-3 high-speed digital camera with CMOS sensor capable of recording at 2000fps at full resolution of 1024 × 1024 pixels with a pixel-size of 17μm, yielding an effective pixel-size of (17μm/px)/337.5 = 50nm/px, which was confirmed experimentally. To reduce memory usage, the required pixel-area was reduced to 256 × 256 pixels, when recording videos of RBCs.

### Preparation of dried RBC

RBCs were extract using the finger pricking method from a voluntary, healthy donor. Approximately 20 − 30μL of blood was aspirated using a pipette and immediately diluted inside 1mL solution containing phosphate buffered saline (Sigma-Aldrich, Germany), 5mM glucose (Riedel-de Haen, Seelze, Germany) and 1mg/mL bovine serum albumin (Sigma-Aldrich, Germany). After extraction the blood is washed with a centrifuge (Mikro 120, Hettich, Germany) (5 × 10 min, 2347.8G). After each centrifugation pass the supernatant is removed and the remaining RBCs are resuspended in 1mL of centrifugation buffer. The RBCs were then incubated for 2 h in a fixation buffer with the same composition as the centrifugation buffer with an added 3% of glutaraldehyde (Sigma-Aldrich, Germany). After incubation, the buffer solvent is exchanged with pure water (Milli-Q) after centrifugation (2 × 10 min, 2347.8G) and substitution of the supernatant. Then, 80μL of the resulting RBC-containing solution is spread on a cover-slide and dried in an oven at 40°C.

**Synthetic images** were calculated following the method proposed by Usenik [21]. We convolute an image containing a circle consisting of 1’s on a background of 0’s with two Gaussians of two different widths *σ*_1_ and *σ*_2_. The radius of the circle was chosen to be 61px, in order to approximately corresponds to the radius of RBC as recorded with our experimental setup, since 61px ∙ 50nm/px = 3μm is roughly the radius of RBC. By subtracting the two resulting images and choosing *σ*_1_ and *σ*_2_ accordingly, we obtain an image with a halo at the position of the circle boundary, which is very similar to that of the experimentally obtained images of the RBCs. Finally, white noise with a Gaussian amplitude distribution was added to the image pixels to simulate the image noise.

**Signal-to-Noise Ratio** of experimentally obtained images is determined by calculating the averaged image intensity 〈*I*〉 and its standard deviation *σ*_*I*_ inside a feature-less ROIs *after* the image corrections has been performed. The SNR definition we user here is then given by SNR = 〈*I*〉/*σ*_*I*_. Note that this definition is different from the typical logarithmic definition used by Usenik (Usenik et al., 2011), where SNR_Usenik_ = 20 log[〈*I*〉/*σ*_*I*_].

## Supporting information

S1 File High-precision flicker spectroscopy contour tracking algorithm: www.github.com/michaelmell/hipfsta. (PDF)

S2 File Containing S1 Fig: Distributions of the height fluctuations of glutaraldehyde-fixed RBC in aqueous buffer; S1 Table: RMSD of the distributions shown in S1 Fig; S2 Fig: Comparison of the spectra obtained from the synthetic dataset; S3 Fig: Fluctuation spectra at different SNR values obtained with the algorithm from reference [13].
(PDF)
