# Supplementary material for: A gradient-based, GPU-accelerated, high-precision contour-segmentation algorithm with application to cell membrane fluctuation spectroscopy
Source: PLoS One. 2018 Dec 6;13(12):e0207376. doi: 10.1371/journal.pone.0207376 (PMC6283589; doi:10.1371/journal.pone.0207376)
Supplement: S1 File — (PDF) [file pone.0207376.s001.pdf]

A gradient-based, GPU-accelerated, high-precision contour-segmentation algorithm with application to cell membrane fluctuation spectroscopy

Michael Mell[a], Francisco Monroy[a,b]

[a]*Mechanics of Biological Membranes and Biorheology, Química Física I, Universidad Complutense, E-28040 Madrid, Spain*
[b]*Translational Biophysics, Instituto de Investigacion Biomédica Hospital Doce de Octubre (i+12), E-28041 Madrid, Spain*

1. Tracking error derivation

Fitting of the linear region of the phase contrast intensity profile in the direction of the local angle φ_j is done using linear regression of the simple linear equation:

$$y_j = b_j \cdot x_j + a_j \tag{SM1}$$

For brevity, we define the background intensity as $\tilde{y}_j = \langle I_j \rangle$. The intercept x_j of the linear regression with the constant background intensity is then given by

$$x_j = \frac{\tilde{y}_j - a_j}{b_j} \tag{SM2}$$

and is used to determine the local coordinate $\vec{p}_j$ as detailed in the main text. Dropping the indices for the local angle j for clarity, we can determine the error of x using error propagation:

$$\sigma_x^2 = \left(\frac{\tilde{y} - a}{b^2}\right)^2 \sigma_b^2 + \left(\frac{1}{b}\right)^2 \sigma_a^2 + \left(\frac{1}{b}\right)^2 \sigma_{\tilde{y}}^2 \tag{SM3}$$

Email addresses: `michael.mell@gmx.net` (Michael Mell), `monroy@ucm.es` (Francisco Monroy)

A linear regression to a set of data points (X_j, Y_j) finds a and b so that the prediction $\widehat{Y} = a + b \cdot X_j$ minimizes the sum of squared errors $\sum(Y_j - \widehat{Y}_j)$ (Kirchner, 2001). The standard error of the slope b is (Kirchner, 2001)

$$\sigma_b = \frac{1}{\sqrt{U}} \frac{S_{X \cdot Y}}{S_X}\,, \tag{SM4}$$

where U is the number of values being fitted, $S_{X \cdot Y}$ is standard deviation of the residuals, and S_X is the standard deviation of X_i. The standard error for the prediction $\widehat{Y}_i$ at some value X_i is given by (Kirchner, 2001)

$$S_{Y_i} = \frac{S_{X \cdot Y}}{\sqrt{U}} \sqrt{1 + \frac{(X_i - \bar{X})^2}{S_X^2}}\,, \tag{SM5}$$

where $\bar{X}$ is the average value of the X_i and S_X^2 is the variance. In our method we evaluate $\widehat{Y}_i$ at the center of the fit range, so that we have $X_i = \bar{X}$ and eq. (SM5) simplifies to

$$S_{Y_i} = \frac{S_{X \cdot Y}}{\sqrt{U}}\,. \tag{SM6}$$

Assuming the standard deviation of the intensity values σ_I is unchanged in the area of the maximal intensity gradient, where we perform the fit, it will be identical to the standard deviation of residuals, so that we can set:

$$S_{X \cdot Y} \equiv \sigma_I \quad \text{and} \quad S_{X \cdot Y} \equiv \sigma_a \tag{SM7}$$

To obtain the average background intensity $\tilde{y} = \frac{1}{T}\sum_{k=1}^{T} I_k$ we average over T intensity values, so that its standard deviation $\sigma_{\tilde{y}}$ is

$$\sigma_{\tilde{y}} = \frac{\sigma_I}{\sqrt{T}}\,. \tag{SM8}$$

Combining eqs. (SM4), (SM6) and (SM7) with eq. (SM3) we obtain:

$$\sigma_x^2 = \frac{\sigma_I^2}{b^2}\left[\left(\frac{\tilde{y} - a}{b}\right)^2 \left(\frac{1}{U \cdot S_X}\right) + \frac{1}{U} + \frac{1}{T}\right] \tag{SM9}$$

The standard error σ_x is necessarily independent of the location at which the intercept is determined and therefore the expected value of x can be set

to 0 without loss of generality. It therefore follows from equation (SM2) that $x = \frac{\tilde{y}-a}{b} = 0$ and eq. (SM9) becomes

$$\sigma_x^2 = \frac{\sigma_I^2}{b^2}\left[\frac{1}{U} + \frac{1}{T}\right] , \tag{SM10}$$

which yields eq. (3) of the main text. Eq. (SM10) was tested numerically with MATLAB by calculating the linear fits, background averages and their intercept in a sufficiently large dataset for each parameter set and determining the standard deviation σ_x of the obtained intercept x.

Note that the U intensity values that are being fitted in this calculation are assumed to be independent of each other. However, the n_{fit} data points used for fitting in the algorithm are not independent of each other due to the linear interpolation, where on average we obtain f_{interp} data points for each pixel of the image. This is the reason, why we set $U = n_{\text{fit}}/f_{\text{interp}}$ when estimating the tracking precision in the main text. The same consideration yields $T = n_{\text{bgkr}}/f_{\text{interp}}$.

2. Tracking precision for glutaraldehyde-fixed RBC in solution

We test our predictions for the tracking precision of RBCs in buffer solution (see Tab. 2, main text) with experimental data obtained from glutaraldehyde-fixed RBCs in buffer-solution. In the main text we used the distribution of the distances $\delta_i = |\vec{P}_i - \vec{P}'_i|$ between corresponding contour coordinates $\vec{P}_i$ of the current and $\vec{P}'_i$ of the previous frame. Here we use a different method and instead look directly at the distributions and root mean square deviation (RMSD) of the radius times series $R(t, \phi)$. Since the membrane fluctuates around its equilibrium position $\langle R(t, \phi)\rangle$, its height above or below the equilibrium position at a given contour angle ϕ and time t is $h(t, \phi) = R(t, \phi) - \langle R(t, \phi)\rangle$. We can then calculate the distribution of the times series $P(h(\phi))$ and its RMSD $\langle h(\phi)^2\rangle$ and average over all contour angles ϕ: $\langle\cdot\rangle_\phi$. In the case of glutaraldehyde-fixed RBC in buffer-solution, which are assumed to not undergo any thermal membrane fluctuation, this should directly yield the positional uncertainty of our tracking algorithm.

Figure SM1 show the average probability distributions $\langle P(h(t, \phi))\rangle_\phi$ for eight glutaraldehyde-fixed RBC. The corresponding values for RMSD are given in table 1 and vary between 1.2nm and 5.2nm with an average of 2.8±1.4nm. As mentioned in the main text, the method used here is sensitive to any drift in focus or diffusion of the cell, since it takes the whole time-series

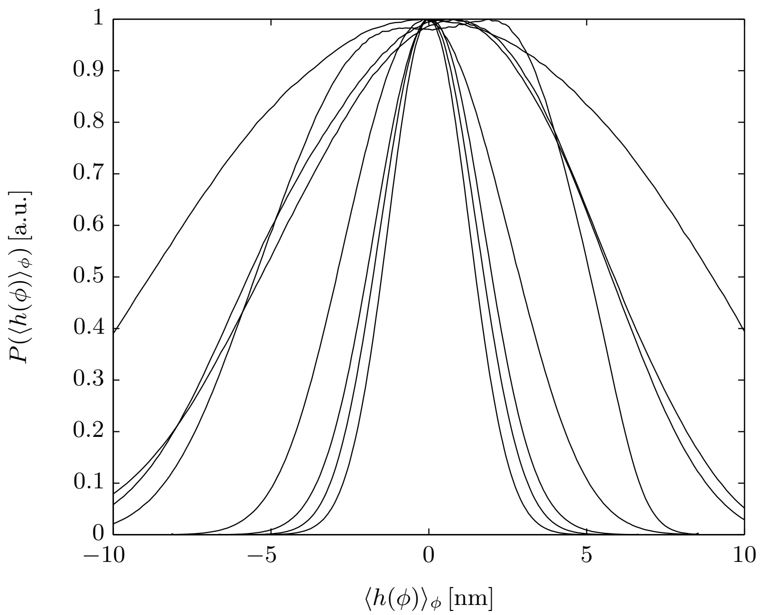

Figure SM1: Distributions of the height fluctuations of glutaraldehyde-fixed RBC in aqueous buffer.

Table 1: RMSD of the distributions shown in Fig. SM1

Dataset	1	2	3	4	5	6	7	8
RMSD	1.2	1.4	1.5	2.2	3.4	3.6	3.6	5.2
⟨RMSD⟩				2.8 ± 1.4				

into account. Since it was found, that glutaraldehyde-fixed RBC exhibit a strong diffusive movement not present in healthy RBC, this movement causes the large dispersion of the determined RMSD values and the non-gaussian and asymmetric shapes of some of the distributions. This is why this method was not used to determine the precision in the main text.

To estimate the precision using this method, we therefore need to focus on the lowest value, which corresponds to cases of glutaraldehyde-fixed RBCs, that did not exhibit appreciable diffusion and had little drift in focus. Averaging over the three lowest RMSD values we obtain an averaged RMSD of 1.3 ± 0.2nm. To compare this to $\delta_{1\sigma}$ from the main text, we calculate $\delta_{1\sigma}^{\exp} = \sqrt{2} \cdot \frac{1.3\text{nm}}{50\text{nm/px}} = 0.037 \pm 0.006$px, which is slightly better than

the predicted value of $\delta_{1\sigma} = 0.052$ from Tab. 2 in the main text for RBC in buffer.

3. Nyquist-Shannon sampling theorem

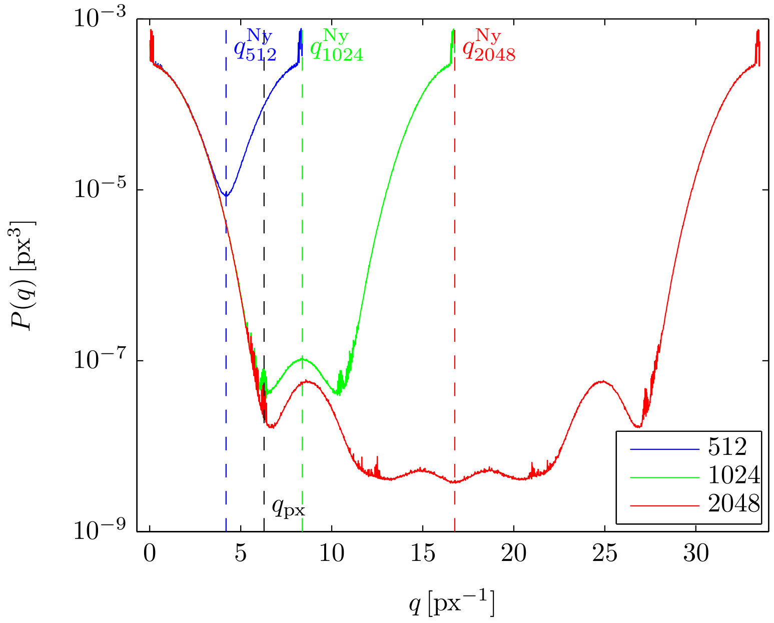

Figure SM2: Comparison of the spectra obtained from the synthetic dataset with SNR = 100 using different numbers of contour coordinates in the tracked contour: $N = 512$, 1024, 2048. When the wavenumber goes above the indicated $q^{\mathrm{Ny}} = N/(2 \cdot R)$, the spectrum is mirrored as a result of the aliasing occurring at wavelengths below the wavelength established by the Shannon-Nyquist sampling theorem. Note that in contrast to the other figures, all units here are in [px]. The simulated contour radius for this simulation was set to $R = 61$px.

The Nyquist-Shannon sampling theorem applies to the Fourier-transform and therefore the spectra of the contour fluctuations obtained from the tracking algorithms. As described in the main text, our algorithm uses N contour coordinates to track and thus *sample* the contour position. These are equally spaced at the contour angles ϕ_i with $\Delta\phi = 2\pi/N$. We can therefore test the validity of the Nyquist-Shannon sampling theorem when applied to our method by changing the number N of contour points used to sample the contour position during tracking.

Figure SM2 shows the spectra obtained from tracking the same synthetic dataset with SNR = 100 and contour radius $R = 61$px with different numbers of contour coordinates $N = 512$, 1024, 2048. Initially all spectra possess nearly identical values up to q^{Ny}_{512}. For $q > q^{\mathrm{Ny}}_{512}$ the spectrum obtained with $N = 512$ starts to increase again and mirrors the values below q^{Ny}_{512}. The same occurs for the spectra obtained with $N = 1024$ and 2048 for $q > q^{\mathrm{Ny}}_{1024}$ and $q > q^{\mathrm{Ny}}_{2048}$ respectively. As explained in the main text, this expected behavior is the result of aliasing resulting from calculating the spectrum at wavelengths $\lambda < \lambda^{\mathrm{Ny}}$, below the wavelength λ^{Ny} established by the Nyquist-Shannon. Taking $N = 2048$ as example we have $q^{\mathrm{Ny}} = N/(2 \cdot R) = 2048/(2 \cdot 61\mathrm{px}) = 16.78\mathrm{px}^{-1}$ and thus $\lambda^{\mathrm{Ny}} = 2\pi/16.78\mathrm{px}^{-1} = 0.374$px. The number of contour coordinates per pixel is on average $2048/2\pi \cdot R = 5.343\mathrm{px}^{-1}$ yielding the inverse sampling wavelength $\lambda_{\mathrm{samp}} = 0.187$px. Thus $\lambda^{\mathrm{Ny}} = 2 \cdot \lambda_{\mathrm{samp}}$ and Fig. SM2 confirms that the Nyquist-Shannon sampling theorem applies to our method.

4. Spectrum analysis of synthetic images using the classical algorithm

To follow up on how the classical algorithm (Pécréaux et al., 2004) performs under different levels of image noise, we determine the spectra for the synthetic datasets with different SNR values as was done in the main text for the new tracking algorithm. Fig. SM3 shows the results.

The noteworthy result here is that the classical algorithm is not able to produce a flat noise floor even at SNR = ∞ – in contrast to the new tracking algorithm (see main text).

We attribute this to the fact that the classical algorithm tracks each contour sequentially, so the value of each contour coordinate $\vec{P}_i$ depends on $\vec{P}_{i-1}$. Furthermore this is also true for the tracking start-position of each image, because the starting position of each image corresponds to the last contour coordinate of the previous image. Therefore each contour coordinate depends on the values of *all* previous contour coordinates of *all* previous images. Therefore the algorithm in unable to converge to a final contour position, which causes residual noise at high spatial frequencies (close to and below the pixel size), even when each of the synthetic images is identical at SNR = ∞.

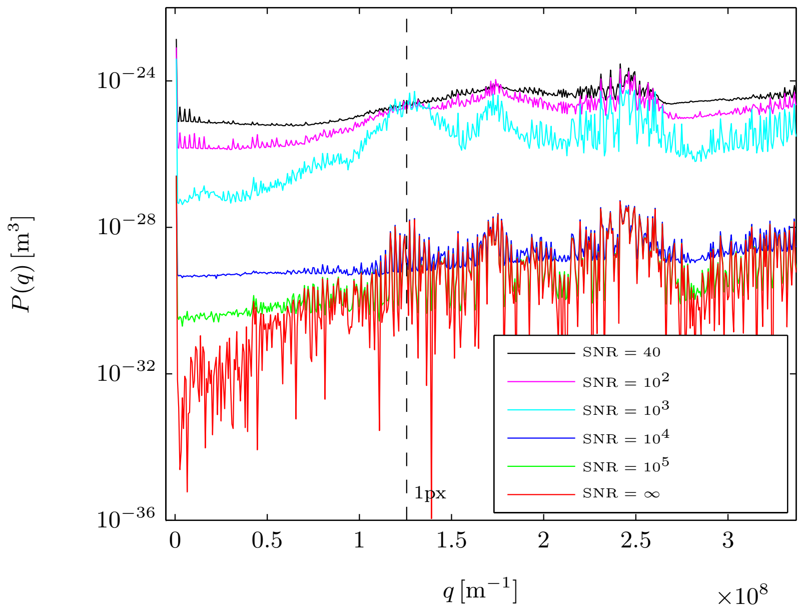

Figure SM3: Fluctuation spectra at different SNR values obtained with algorithm the algorithm from (Pécréaux et al., 2004).

Kirchner, J., 2001. Simple linear regression. URL http://seismo.berkeley.edu/~kirchner/eps_120/Toolkits/Toolkit_10.pdf

Pécréaux, J., Döbereiner, H. G., Prost, J., Joanny, J. F., Bassereau, P., 2004. Refined contour analysis of giant unilamellar vesicles. The European Physical Journal E 13 (3), 277–290.
